# Enhanced enrichment of extracellular vesicles for laboratory and clinical research from drop-sized blood samples

**DOI:** 10.3389/fmolb.2024.1365783

**Published:** 2024-08-15

**Authors:** Alexa Guerrero-Alba, Sandhya Bansal, Aryan N. Sankpal, Geetanjali Mitra, Mohammad Rahman, Ranjithkumar Ravichandran, Christin Poulson, Timothy P. Fleming, Michael A. Smith, Ross M. Bremner, T. Mohanakumar, Narendra V. Sankpal

**Affiliations:** Norton Thoracic Institute, St. Joseph’s Hospital and Medical Center, Phoenix, AZ, United States

**Keywords:** EVS, exosome, marker, blood, transplant, diagnosis

## Abstract

In the realm of biomedical advancement, extracellular vesicles (EVs) are revolutionizing our capacity to diagnose, monitor, and predict disease progression. However, the comprehensive exploration and clinical application of EVs face significant limitations due to the current isolation techniques. The size exclusion chromatography, commercial precipitation reagents, and ultracentrifugation are frequently employed, necessitating skilled operators and entailing challenges related to consistency, reproducibility, quality, and yields. Notably, the formidable challenge of extracellular vesicle isolation persists when dealing with clinical samples of limited availability. This study addresses these challenges by aiming to devise a rapid, user-friendly, and high-recovery EVs isolation technique tailored for blood samples. The NTI-EXO precipitation method demonstrated a 5-fold increase in the recovery of serum EVs compared to current methodologies. Importantly, we illustrate that a mere two drops of blood (∼100 µL) suffice for the recovery of enriched EVs. The integrity and quality of these isolated EVs were rigorously assessed for the size, purity, and contaminants. This method was validated through the successful isolation of EVs from organ transplant recipients to detect disease-specific exosomal markers, including LKB1, SARS-CoV-2 spike protein, and PD-L1. In conclusion, NTI-EXO method can be used for small clinical samples, thereby advancing discoveries in the EV-centric domain and propelling the frontiers of biomedical research and clinical applications.

## Introduction

Extracellular vesicles (EVs) represent a fundamental component of intercellular communication, being secreted by numerous cell types. These vesicles, present in various biological fluids, encapsulate functional proteins, metabolites, and nucleic acids originating from their host cells (Chaput and Thery, 2011; Nieuwland et al., 2018, Falcon-Perez et al., 2018). Recent research endeavors have yielded significant insights into the potential diagnostic and monitoring roles of EVs in disease pathology (van der Pol, 2012; van Niel et al., 2018). The International Society for Extracellular Vesicles (ISEV) has introduced guidelines (MISEV 2018 and MISEV 2023) to establish standards for extracellular vesicle (EV) research. EVs, which include small EVs (<200 nm) and medium/large EVs (>200 nm), are frequently of interest in research (Thery et al., 2018; Welsh et al., 2024). The scientific community’s focus on EVs stems from their distinctive capacity to facilitate cellular communication while transporting a payload of proteins and nucleic acids, thereby governing diverse biological and pathological processes (Meckes et al., 2010; Yanez-Mo et al., 2015).

Detection of disease-specific biomarkers from isolated EVs is rapid, practical, and effective. Isolated EVs have the potential to provide early diagnosis of diseases such as cancer from bodily fluid like a liquid biopsy or a minimally invasive blood draw (Li, Yi et al., 2021). Notably, it has been shown that EVs carrying cancer specific proteins and RNA that promote cancer progression (Abd Elmageed, Yang et al., 2014; Le, Hamar et al., 2014; Melo, Sugimoto et al., 2014). Moreover, the ability of EVs to carry a diverse cargo load holds promise for therapeutic drug delivery (see current clinical trials NCT01294072, NCT04879810, NCT02657460 and NCT01854866). Such advances demonstrate promising alternative therapies for patients with Parkinson’s (Kojima, Bojar et al., 2018), cardiovascular, and chronic kidney (Nassar, El-Ansary et al., 2016) disease. The emerging evidence illuminating the significant contributions of EVs to pathological conditions, including but not limited to cancer, organ transplant rejection, autoimmune disorders, neurological diseases, and infections, has garnered substantial attention among researchers (Li, Man et al., 2021; Campos-Mora, De Solminihac et al., 2022; Yates, Pink et al., 2022). Our previous studies in lung transplant models have shown that EVs may be useful to monitor allograft-related immune responses (Bansal, Sharma et al., 2018; Sharma, Ravichandran et al., 2018, Bansal, Limaye et al., 2021) and lung allograft rejection (Gunasekaran, Sharma et al., 2018; Ravichandran, Bansal et al., 2019).

EVs capitalize on notable attributes such as high stability, low immunogenicity, target specificity, and biocompatibility. Nonetheless, the methods employed for EV recovery and enrichment currently exhibit significant variability across different laboratory settings (Witwer, Buzas et al., 2013; Taylor and Shah, 2015), limiting standardization and large-scale production. There are many EV isolation methods, but those that promote both EV integrity and purity are limited. Ultracentrifugation and size exclusion chromatography are the most common isolation methods but are limited due to low yield (Thery et al., 2006). Other practices commonly used for EV isolation are immune-affinity capture-based techniques, precipitation reagents, and microfluidic-based methods. However, these methods also possess challenges such as cost, loss of structural integrity, and contamination (Li, Kaslan et al., 2017; Doyle and Wang, 2019).

Although EVs can be isolated from various bodily fluids, blood is commonly used in clinical research, volume sufficiency for experiments is still challenging. On average, research that involves EV isolation from cell cultures utilizes an excess volume of media to obtain an adequate quantity of EVs (Veerman, Teeuwen et al., 2021). Similarly, when working with breast milk, urine, and other biological fluids, a large starting volume is required (Gardiner, Di Vizio et al., 2016). Therefore, a crucial limiting factor that impacts almost all current isolation methods is small or dilute samples (Kalluri and LeBleu, 2020).

In the past, our laboratory has utilized several EV isolation methods for studies and often seen inconsistent EV recovery, which varies from sample to sample (Nassar, El-Ansary et al., 2016; Bansal, Sharma et al., 2018; Sharma, Ravichandran et al., 2018; Bansal, Limaye et al., 2021). This, combined with the scarcity of precious patient serum and plasma samples, limited our studies (Ravichandran, Bansal et al., 2019; Bansal, Limaye et al., 2021). To this end, we have developed a more reliable method, NTI-EXO, which we have compared to several established methods, including size exclusion chromatography (SEC), ultracentrifugation (UC), and precipitation with commercially available reagents. In a stepwise isolation process succeeded by comprehensive characterization, we substantiated the reliability of this approach using diverse fresh and frozen serum as well as plasma samples from both animal and human subjects. We anticipate that the NTI-EXO method will serve as a valuable asset for the EV research community and ongoing endeavors in EV marker discovery.

## Materials and methods

### Cell culture

Human embryonic kidney 293T cells (ATCC CRL-11268™) were cultured in Dulbecco’s Modified Eagle’s Medium (DMEM; Hyclone #SH30243)10% fetal bovine serum (FBS; R&D System #S11550).H1299 cells (ATCC, CRL5803) were cultured in RPMI (Hyclone #SH30027) supplemented with 10% FBS and antibiotics PenStrep. Cultures were incubated at 37°C in with 5% carbon dioxide in humidified conditions.

### Patient and animal serum or plasma collection

Yorkshire porcine blood was collected from an ongoing study during different time points of an organ care system experiment at our facility. The porcine blood samples were collected from the recycled reservoir of the organ care system, processed for serum separation, and stored at −20°C. Human serum was procured from Sigma-Aldrich (Cat #H4522). Patient blood samples were collected in sodium heparin tubes. Blood was collected from mice by puncturing the sinus to collect one to three drops of blood. Collected blood was centrifuged at 2000 *g* for 10 min to separate the serum/plasm followed by storage or EV extraction. The approved methods was used to anesthetize, sacrifice and blood collection for mice. Briefly, animals were anesthetized with 87 mg/kg of a Ketamine/Xylazine cocktail, the fur was clipped and the animal was restrained followed by passively administration of 1.0% Isoflurane at 1L/min. After verifying the level of anesthesia is sufficient, a 3 cm abdominal midline incision was made. The Inferior Vena Cava was visualized and the animal was exsanguinated to collect approximately 0.8 mL of blood. Mice were scarificed by cervical dislocation. All experimental protocols were approved by the Institutional Review Board of St. Joseph’s Hospital and Medical Center committee. All methods were performed in accordance with the relevant Institutional guidelines and regulations. All methods are reported in accordance with ARRIVE guidelines (https://arriveguidelines.org).

### EV isolation, NTI-EXO precipitation

**FIGURE 1:**
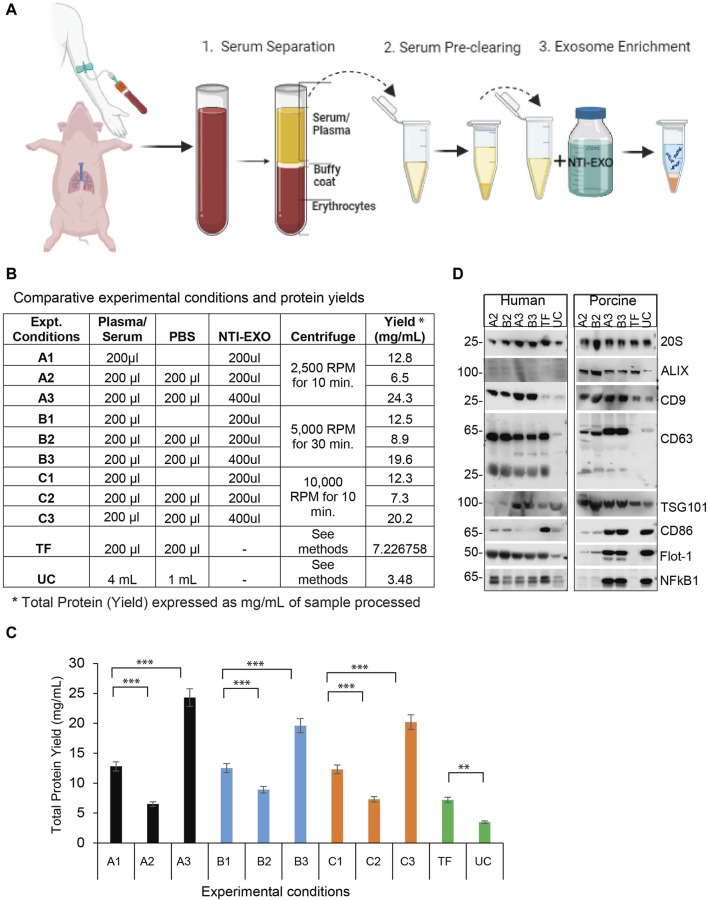
A comparison of NTI-EXO experimental conditions for the efficient recovery of EVs from human and porcine serum **(A)** A depiction of the NTI-EXO protocol yielding EVs from human and porcine serum. **(B–C)** EVs yields isolated from normal human serum from various combinations of NTI-EXO, PBS, and centrifugation speeds. **(D)** Characterization of human- and porcine-derived EVs using western blot. EVs recovered by NTI-EXO, TF (Thermo Fisher reagent), and UC (ultracentrifugation) were compared. CD9, CD63, CD86, ALIX, 20 s, Flot-1, NFkB1, and TSG101 were analyzed. Some of these markers were better enriched with the NTI-EXO method than commercial or standard (UC) methods. Twenty micrograms of the total protein were used for immunoblotting experiments. Experimental conditions A1-A3, B1-B3 and C1-C3 are individual isolation conditions. Values are given as mean ± S.D. of three independent experiments. Statistical significance was defined at **p < 0.01, and ***p < 0.001 compared to the corresponding control.

The NTI-EXO reagent was prepared using 22.5% polyethylene glycol (PEG) with Mn (average molecular weight) of 6,000 (Sigma, 81,260), buffered with sodium chloride 0.2 M, EDTA 10 mM, and 200 mM Tris-pH 7.2. The NTI-EXO solution was filtered through a 0.2-micron filter and stored up to a year at 4°C. EV isolation from separated serum was carried out in two steps: preclearing and EV precipitation. For the preclearing step, blood was centrifuged at 2000 *g* for 10 min. Cell-free samples (serum or plasma) were transferred to a new Eppendorf tube and centrifuged at 4°C on a benchtop Eppendorf centrifuge (5417R) at 10,000 RPM for 30 min. These precleared serum or plasma samples were used for EV isolation as depicted in Figure 1. For the EV precipitation step, in a standard optimized protocol, 200 µL of PBS was added to 200 µL of precleared plasma or serum together with 400 µL of the NTI-EXO reagent. For the low pH NTI-EXO-Acetate method, 5 µL of 1 M Na-acetate, pH 4.5, was added along with 60 µL of 5 M NaCl to 100 µL of serum. Tubes were rotated at 4°C for 30 min then centrifuged at 2500 RPM for 10 min. The supernatant was gently aspirated out, and pellets were gently washed once with 500 µL of PBS, re-suspended in 200 µL of PBS and vortexed for 10–15 s. Samples that did not have a fully solubilized pellet were kept at 4°C overnight followed by centrifugation to obtain clear solution. All isolated EVs were assayed for total protein concentration using a BCA Protein Assay kit (#23227, Thermo Scientific).

Commercial EV isolation was performed using the Total EV Isolation Reagent (from serum) as recommended by the manufacturer (#4478360, Thermo Fisher Scientific). Briefly, the EV isolation reagent was mixed with serum and incubated for 30 min at 4°C and centrifuged at 10,000 g to obtain an EV pellet. The pellet was dissolved in PBS and centrifuged at 12,000 g and passed through a 0.22-µm filter to obtain pure EVs.

### EV isolation, ultracentrifugation

For preclearing, serum samples were centrifuged at 10,000 rpm for 60 min at 4°C in a Beckman Coulter Optima XE-90 Ultracentrifuge with a 70.1 TI Fixed-Angle Titanium Rotor (Beckman Coulter, Ser.#1851). The supernatant was then transferred into new tubes and ultracentrifuged at 100,000 g for 2 h at 4°C. The supernatant was removed, and the pellets were washed with PBS and centrifuged at 100,000 g for 30 min at 4°C. PBS wash was removed, and the recovered pellets were air dried and re-suspended into PBS. Cell culture based EVs isolation was performed as described earlier (Rahman, Ravichandran et al., 2023).

### EV isolation, size exclusion chromatography

The SEC method has been previously described (van der Pol, 2012; Ter-Ovanesyan, Norman et al., 2021). Briefly, serum preparations were precleared to remove micro-vesicles. Ten mL of Sepharose CL-2B (#CL2B300, Sigma-Aldrich) resin was loaded into a 20-mL Bio-Rad column and washed with PBS, then 1.0 mL of serum was loaded followed by elution and collection of 1.0 mL or 0.5 mL fractions. Collected fractions were assayed for total protein content.

### Nanoparticle tracking analysis

The NanoSight was primed by running 5 mL of Invitrogen Ultra-Pure DNase/RNase-free distilled water using a 1 mL syringe. Isolated EV samples were diluted into PBS at a 1:100 ratio. A 500 µL sample was run through NanoSight capturing three, 30-s videos. Nanoparticle tracking analysis (NTA) software then analyzed footage at a detection threshold of 5 based on EV size. EVs that were <200 nm in diameter per 2018 Minimal Information for Studies of Extracellular Vesicles [MISEV] guidelines) were used as pure EVs (Thery et al., 2018). The NanoSight software version 3.3 (Malvern, UK) was used to analyze the data.

### ExoView analysis

EVs isolated from human, porcine, and mouse samples using NTA-EXO or SEC were characterized for total and co-localization detection of CD81, CD63, and CD9 markers together with an isotype control, MIgG. ExoView R200 platform (ExoFlex Tetraspanin chips, NanoView Biosciences, Boston, MA) was used as recommended by the manufacturer. Briefly, ExoView Tetraspanin chips were placed onto a 24-well plate and pre-scanned utilizing the ExoView software. Using the ExoFlex Human Tetraspanin Kit, samples were prepared with 1X incubation solution II in a 1:10 ratio. Fifty microliters of diluted sample were loaded onto the plate with the pre-scanned chips and incubated at room temperature overnight. Following incubation, 1 mL of 1X Solution A was added to each well, washed on an orbital shaker at 500 RPM for 3 min, and aspirated. Chip washing steps were followed for 3 cycles. After the final wash, 250 µL of fluorescently labeled antibody mixture of anti-CD9, anti-CD81, and anti-CD63 was added to each well. The plate was then covered with foil to ensure all light was blocked and placed on the orbital shaker for 1 h at 500 RPM. After antibody incubation, chips were washed 3 times with 1 mL 1X solution A I, 1X solution B I, and distilled water. EV-coated chips were analyzed using the ExoView R200 reader as recommended by the manufacturer. Data was analyzed using ExoViewer 2.5.0 with the size threshold set to 50–200 nm in diameter. Normalized particles were counted as the differences between pre- and post-scan.

## SDS-PAGE Coomassie blue staining

Isolated EVs were mixed with sample buffer 1X Laemmli buffer (2%SDS, 10 mM EDTA, 10% Glycerol and 2.5%-mercaptoethanol,120 mM Tris-HCl, pH6.8) boiled for 10 min. For total protein detection, 6 μg of total protein was loaded onto a NuPAGE 4%–12%, Bis-Tris gradient gel. The gel was placed in a fix solution (50% ethanol, 10% acetic acid, and 40% Milli-Q water) for 10 min, followed by 0.5% Coomassie blue G-250 staining for 10 min. After de-staining, the gel was placed onto a white background and imaged.

### Western blot

Isolated EVs were mixed with to 1X Laemmli buffer to a final protein concentration of 2 μg/μL and boiled for 10 min. Twenty micrograms of EVs solution were loaded onto an Invitrogen NuPAGE 4%–12%, Bis-Tris gel, then transferred to a polyvinylidene difluoride (PVDF) membrane, rinsed with Milli-Q water, and blocked with 5% non-fat dry milk (#1706404, Bio-Rad). 1X Tris-buffered saline with 0.1% Tween-20 (TBST) was used as a wash and antibody diluent buffer. Primary antibodies used for the study were: CD9 (#312102; BioLegend), CD63 (#25682-1; ProteinTech), CD80 (#66406-1; ProteinTech), CD81 (Cell Signaling), flotulin-1 (#orb18698; BioOrbit), proteasome 20 s (#sc-271187; Santa Cruz), NFKB (ProteinTech), Alix (#ab88388; AbCAM), TSG101 (#ab83; AbCAM), CD63 (#sc-365604; Santa Cruz Biotechnology), PD-L1 (#ab213480; Abcam), CD73 (#D7F9A; Cell Signaling Technology), ApoA1 (#3350S, Cell Signaling), Albumin (#4929S, Cell Signaling) and kappa light chain HRP (#A18853; Thermo Fisher Scientific). Following wash of primary antibodies, the appropriate secondary HRP-conjugated antibodies were used. All blots were revealed using SuperSignal West Pico PLUS Chemiluminescent Substrate (Cat# 34580, Thermo Fisher Scientific). Chemiluminescent signals were acquired with the Odyssey-Fc Imager (LI-COR).

### Transmission electron microscopy (TEM)

Isolated exosome pellet was re-suspended in PBS followed by particle measurement using NTA and protein measurement. NTA measurement shows exosome counts 10X^7^ per ml. In two steps, samples were prepared to acquire images. Fixation to preserve EV morphology was done by fixing of EVs in 1 mL of 2.5% glutaraldehyde for 1 h at 4°C. Fixed EVs pellet was washed three times with 0.5-mL PBS and re-suspended in 200-µL PBS. Adsorption and negative staining of EVs to a TEM grid was done by pipetting EVs onto glow-discharged 200 mesh carbon-coated copper EM grids (Electron Microscopy Sciences), allowed to briefly drying before negative staining with 3% phosphotungstic acid. Grids were stained for approximately 1 min before rinsing in ultrapure water and allowed to dry. Images were acquired on a Talos L120C transmission electron microscope operating at 120 kV at Arizona State University’s Eyring Materials Center.

### EVs reporter cell line generation for integrity and uptake assay

Dual-color fluorescent reporter cell line expressing CD63-positive EVs was generated using pLenti-pHluorin_M153R-CD63-mScarlet plasmid (addgene #172118). Viral particles were generated as described before (Sankpal, Brown et al., 2021). Briefly, viral particle containing medium was harvested after 48-h post-transfection. After filtration through 45-micron filter, medium was supplemented with polybrene (Sigma, #9268) at 10 μg/mL final concentration and added to H1299 cells at 10 MOI. After confirming >90% positive cells for GFP and RFP, H1299 cells were cultured in 10-CM dish. Cells were grown in 10% FBS-DMEM media, washed with PBS two times and culture media was replaced with 10-mL Optimem, incubated for 48-h. The 10-mL conditioned medium was harvested, precleared and ultracentrifuged to purify reporter exosomes as described in earlier section. The purified exosomes pellet was re-suspended in 500 µL of PBS. 20µL of EVs was added to a well 12 well-cultured NIH-3T3 cells as UC-exosomes. The 200 µL of the two aliquots were added to two-200 µL exosome free serum tubes processed for exosome isolation using commercial TF and NTI-EXO method. Washed pellets were re-suspended in 200 µL PBS and 50-ul of the reporter exosomes were added to NIH-3T3 cells cultured in 12-well plate containing 0.5 mL culture media. After overnight incubation, followed by washing exosomal uptake was imaged using EVOS M7000 imaging system (ThermoFisher Scientific) using 20X objective at GFP and RFP channel.

### Statistical analysis

All experiments were performed at least 3 times. Statistical analyses were performed using GraphPad Prism 9.4 (GraphPad, La Jolla, CA). Numerical data are presented as mean ± standard deviation. Single comparisons were performed by unpaired Student’s t tests. Evaluations were performed using a one-way nonparametric test, the Kruskal–Wallis method. The comparison of two groups was performed by using the Mann–Whitney’s U test. *p*-values <0.05 were considered statistically significant.

## Results

### Isolation of EVs from human and porcine serum using NTI-EXO

To develop NTI-EXO, an EV isolation method we used normal human and porcine serum samples. Experimental parameters such as serum dilution, NTI-EXO reagent concentration, incubation time, and centrifugation speed were used as variables. Figure 1A depicts the overall 3-step, 1-h protocol to isolate EVs. For comparative studies, we included UC and a commercial precipitation method (Thermo Fisher [TF]) to compare overall recovery, yields, and quality of EVs. Final recovery of proteins was measured as mg/mL, and quality and concentration of EVs were characterized using NanoSight and SDS-PAGE. From repeated experiments, we found that experimental conditions A3, B3, and C3 provided better recovery conditions than the others (Figure 1B). From 200 µL of serum we recovered 5 mg (SEM ± 0.4) of total protein using experimental conditions A3 and B3 (Figure 1C). Applying conditions A2 and B2 to human serum showed lower yields but somewhat higher purity based on western blots (Figure 1D). However, A3 and B3 isolation methods were highly reproducible when using normal human and porcine serum samples. Thus, we used A3 (lower centrifugation speed 2500 RPM) as the optimized NTI-EXO isolation method throughout the studies. Loading 20 µg of the isolated EV-enriched proteins was sufficient to detect EV markers from human and porcine samples (Figure 1D). Using samples collected from multiple porcine transplant experiments, we consistently observed enriched EVs detected by markers including CD9, CD63, CD81, ALIX, TSG101, 20S, NF-kB and Flot-1.

**FIGURE 2:**
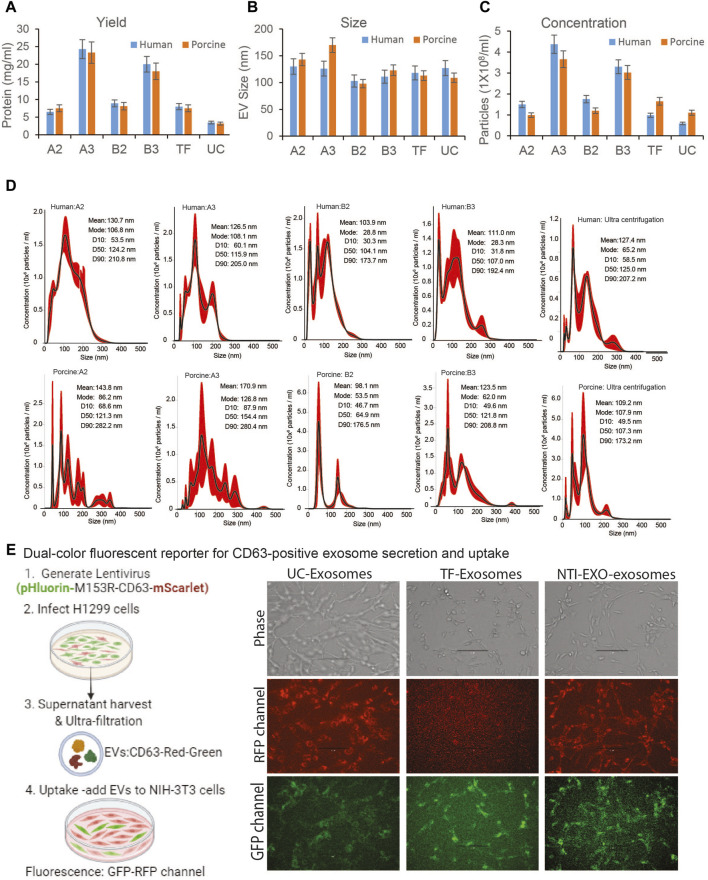
Comparative characterization of EVs isolated from human and porcine serum using NTI-EXO or ultracentrifugation. **(A)** Yields, **(B)** size, and **(C)** concentration of EVs isolated by different experimental conditions (see Figure 1B). Total protein yields were assayed by BCA; Size and concentration were analyzed using NanoSight data (NTA). **(D)** NanoSight plots displaying EV size and calculated mean, mode, and size characterization. **(E)** Cartoon showing overall EVs uptake assay. EVs were added to NIH-3T3 cells; UC-exosomes (reporter exosomes isolated using ultracentrifugation), TF-Exosomes (commercial kit isolated exosomes) and NTI-EXZO isolated exosomes. Statistical significance was defined at **p < 0.01, and ***p < 0.001 compared to the corresponding control.

Next, the quality, size, and concentration of isolated EVs was determined using NanoSight (NTA). As anticipated, experimental conditions A3 and B3 proved to be the best for overall recovery and particle concentration (Figures 2A,C). NanoSight data also suggested that conditions A3 and B3 produced a cleaner population of EVs ranging in size from 110 to 120 nm than other conditions (Figures 2B,D; Supplementary Figure S2). Detailed analysis suggested that these enriched EV’s with a mean particle diameter of 100 ± 20 nm were free of contaminating particles. This enrichment of a clean EV population also correlated with the presence of EV markers (Figure 1D). More strikingly, A3 and B3 experimental conditions demonstrated better detection of porcine EV markers CD9, CD63, Flot-1, CD86, and NF-kB than commercial precipitation or UC methods. Next, the quality of EVs isolated by NTI-EXO was compared to the gold standard, size exclusion chromatography (SEC). SEC is known to purify EVs with high integrity with minimal alterations (Gamez-Valero, Monguio-Tortajada et al., 2016). Some fractions of EVs isolated by SEC carried specific markers CD9, CD63, TSG101, and NF-kB1 (Supplementary Figure S1). However, apart from the advantage of purity, SEC was less efficient and more time consuming and generated a 10-fold increase in downstream volume compared to the NTI-EXO method.

To validate integrity of the NTI-EXO isolated vesicles, we generated stable cell line producing EVs with green and red fluorescent proteins fused to CD63 (Sung, von Lersner et al., 2020). Generated lentiviral particles were added to H1299 producer cells with high transduction avility. Confluent culture of H1299 cells was switched to serum-free media for 48-h after PBS wash. Fluorescent vesicles were isolated using ultracentrifugation, pellet was re-suspended in PBS and added to exosome free serum followed by EV isolation using TF and NTI-EXO methods. For uptake assay, 100-µg of fluorescent vesicles were added to NIH-3T3 cells cultured in 12-well plate. After overnight incubation, cells were washed and imaged at RFP and GFP channel. As shown in Figure 2E, right panel; UC isolated EVs were very efficient in uptake by HIN-3T3 cells as seen at RFP and GFP channel. TF-EVs were poor in uptake as seen in RFP channel. We also observed some auto-fluroscence at GFP channel. In comparison to UC-EVs, NTI-EXO isolated fluroscent EVs shows satisfactory results demonstrating that our method has enriched EVs with overall good integrity.

### High-yield EV precipitations along with albumin and immunoglobulins did not interfere with downstream analysis

**FIGURE 3:**
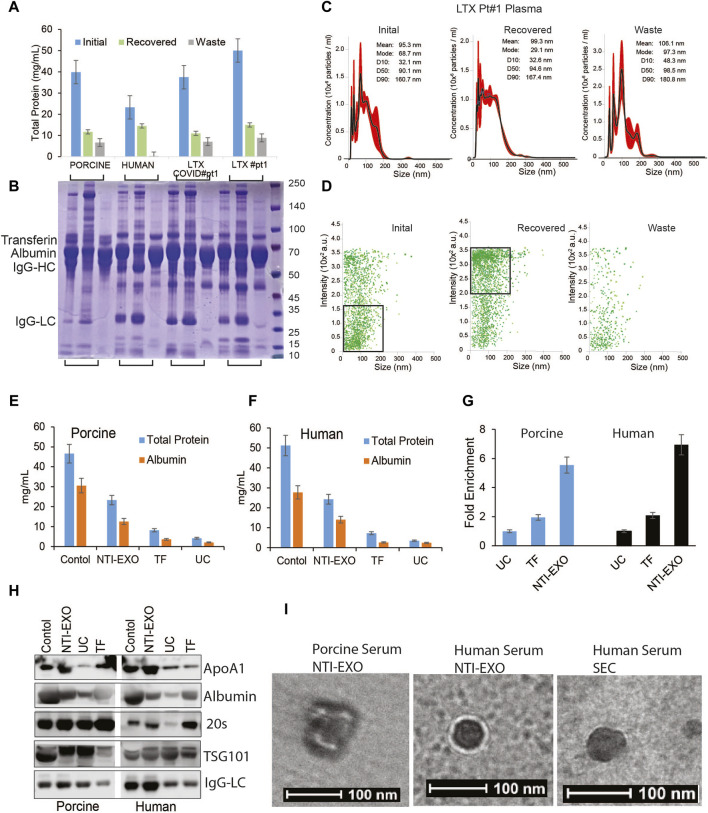
Exosomal protein recovery and characterization from serum samples. **(A, B)** Total initial, recovered, and not recovered (waste) protein yields from NTI-EXO of porcine and normal human serum samples and 2 serum samples from lung transplant patients. Total isolated EV-enriched protein was analyzed using BCA and SDS-PAGE-Coomassie. **(C, D)** NanoSight data demonstrating distribution of EVs. Results show that based on initial plasma proteins, 20%–30% of EV-enriched proteins were recovered. Based on concentration, over 95% of EVs were isolated from 4 different samples. **(E, F)** Control serum and EVs isolated using NTI-EXO, TF or UC were analyzed for total protein and albumin (BCG assay). **(G)** Fold enrichment of total protein is compared in three methods for human and porcine serum. **(H)** EVs purity was further assayed for initial and carried over albumin and ApOA1 using immunoblotting. **(I)** Whole mount TEM images of EVs enriched using NTI-EXO precipitation of human and porcine serum and SEC. Scale bars, 100 nm. Statistical significance was defined at **p < 0.01, and ***p < 0.001 compared to the corresponding control.

Compared to previously reported yields, the higher protein yields recovered by NTI-EXO raised a question about purity of the precipitated EVs. The major proteins in serum are albumins (50%–60%), globulins (40%), immunoglobulins (10%–20%), and miscellaneous other proteins, which yields 60–80 mg/mL of total protein (Leeman, Choi et al., 2018). To investigate the contents of the purified samples, we used normal human serum, normal porcine serum, and serum from 2 human lung transplant patients, one with COVID-19 and one without COVID-19. These 4 samples (200 µL each) were subjected to EV isolation using condition A3 (Figure 1B). The protein concentration of cleared serum (initial), NTI-EXO isolated (recovered), and NTI-EXO not precipitated (waste) was determined. As shown in Figure 3A, the NTI-EXO A3 condition recovered 20%–30% of the total protein from the initial samples. Six micrograms each of the initial, recovered, and waste proteins were resolved on SDS-PAGE followed by Coomassie blue protein staining. As shown in Figure 3B, albumin (66 kDa), IgG heavy chain (50 kDa), and IgG light chain (25 kDa) were clearly observed along with other proteins in initial samples. Interestingly, when compared to initial serum, the recovered samples had less albumin and IgG heavy chain, which was found in the waste. The recovered samples were analyzed for particle size and concentration using NanoSight. For example, the recovered EVs in clinical sample LTX#1 produced a single peak ∼100 nm in size (Figure 3C). To add, a scatter plot demonstrates a high-intensity EV population or efficient enrichment in recovered samples compared to the waste (Figure 3D). Altogether, these results from 4 different samples demonstrate that EVs isolated using the NTI-EXO method are recovered efficiently with minimal albumin and IgG. Throughout the experiments, these carried over proteins did not interfere with EV characterization or assays. To analyze major contaminating protein from isolated EVs, we assayed total protein and major contaminant albumin. Colorimetric assay shows that in control sera has total protein to 45 and 50 mg/mL for porcine and human respectively. EVs isolated from using NTI-EXO, TF and UC methods still shows similar ratio of total protein to albumin (Figures 3E,F). NTI-EXO isolated total protein recovery was 23 mg/mL of which 11 mg/mL was albumin. Total protein for TF and UC was 6.6 and 3.2 mg/mL. The compiled data when analyzed for fold yields as protein, TF yields were 2-fold and NTI-EXO yields were more than 5-fold for porcine and human (Figure 3G). Further validation was carried out using 20 μg of the total protein for immunoblotting analyzed for exosome markers, 20 s, TSG101 and lipoprotein marker ApoA1 and albumin. In spite of 5-fold higher recovery of NTI-EXO method total albumin contamination remained same for porcine and human sera samples (Figure 3H). The isolated EVs were further subjected for TEM analysis for size, purity determination. Negatively stained round particles were seen at around 100 nm similar to SEC isolated EVs (Figure 3I). The overall results demonstrates that UC, TF and our NTI-EXO method to purify EVs together with contaminating proteins.

### NTI-EXO improved recovery and isolation of custom-sized EVs

**FIGURE 4:**
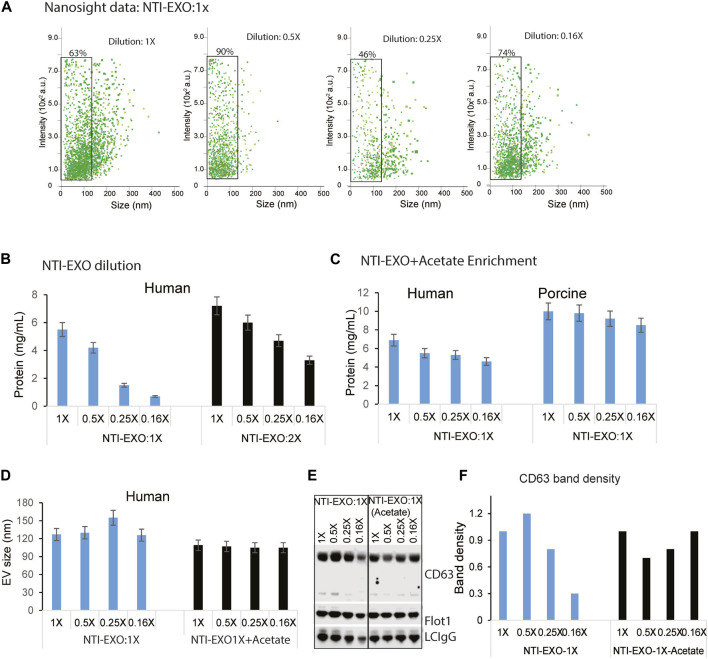
Optimal NTI-EXO concentration to enrich EV isolation. The NTI-EXO method was used to isolate EVs under 100 nM in diameter. The NTI-EXO reagent at a ratio to final volume of 1X, 0.5X, 0.25X and 0.16X was used. **(A)** NanoSight detection of EV intensity *versus* size of the population. **(B)** Protein concentration of adding the reagent in a 1X ratio (blue) *versus* 2X ratio (black) was measured by BCA **(C)**. Lowering pH with a low concentration of NTI-EXO improved EV recovery with EVs size at 120 nm **(D)**. Sodium acetate pH 4.5 was added with NTI-EXO 1X, EV size measured ny NTA. **(E, F)**. Western blots data demonstrates that NTI-EXO dilution reduced EV recovery, which can be compensated for by lowering the pH. This recovery was confirmed by immunoblotting CD63 and Flot-1 together with a loading control, LC-IgG (kappa light chain IgG). Band intensity was analyzed and plotted using ImageJ software. Statistical significance was defined at **p < 0.01, and ***p < 0.001 compared to the corresponding control.

Several EV isolation methods including Extra-PEG (Rider, Hurwitz et al., 2016), combined PEG-UC (Ludwig, De Miroschedji et al., 2018), and the commercial method ExoQuick (System Biosciences) are based on the same principle of particle precipitation. However, none of these methods has the ability to restrict the size of EVs. Most of the PEG precipitation methods, including UC and NTI-EXO, isolate particles ranging from 50 to 250 nm (Figure 2D; Supplementary Figure S2). To explore if the NTI-EXO method could enrich certain sized EVs, we used NTI-EXO at 2X as the stock solution with further dilutions to 1X, 0.5X, 0.25X and 0.16X. Since PEG precipitation of EVs is concentration dependent, we anticipated that more dilution would recover less EVs. Of note, for our initial optimization experiments, we used a serum:NTI-EXO ratio of 1:2. To our surprise, NTI-EXO:1X at 0.5X dilution enriched 90% of gated EVs under 100 nm when compared to 1X, 0.25X and 0.16X dilution conditions (Figure 4A, gated population). These results suggest that with careful experimental design, a clean population of size-restricted EVs can be isolated. As shown in Figure 4B, serial dilution of NTI-EXO:1X decreased total protein yields, which was improved by 20% with NTI-EXO:2X.

Encouraged by restricting the size of EVs, we also attempted to isolate EVs by altering the NTI-EXO method. An acetate-based salting out method was designed to isolate EVs from culture media at an acidic pH (Brownlee, Lynn et al., 2014). In an experimental setup, a serial dilution of NTI-EXO:1X was used with and without acetate. As anticipated, the yield of EVs isolated using only NTI-EXO:1X decreased as the dilution factor was increased. However, acetate-spiked NTI-EXO:1X enrichment of EVs provided more than 30% higher yields without any significant decrease as the dilution was increased (Figure 4C). It was intriguing to observe if acetate spiked EVs purification was of different size. As shown in Figure 4D, human serum subjected for NTI-EXO enrichment shows EVS size 130–150 nm, however TNI-EXO spiked with acetate shows consistent 100 nm EVs size even at different NTI-EXO dilutions. The NTI-EXO-acetate enrichment of EVs (rather than other proteins) was verified using immunoblotting. Even though 20 µg of total protein was loaded, the band densities of CD63, Flot-1, and the loading control LC-IgG decreased with increased dilution of NTI-EXO:1X. However, the NTI-EXO-acetate method maintained CD63, Flot-1, and LC-IgG band density with increased dilution (Figures 4E,F). These results suggests that the NTI-EXO method can be further improved for better recovery. We strongly believe that further studies are necessary to optimize isolation of custom-sized EVs for specialized applications or assays.

### Size, integrity, and EV marker distribution of NTI-EXO-isolated EVs

**FIGURE 5:**
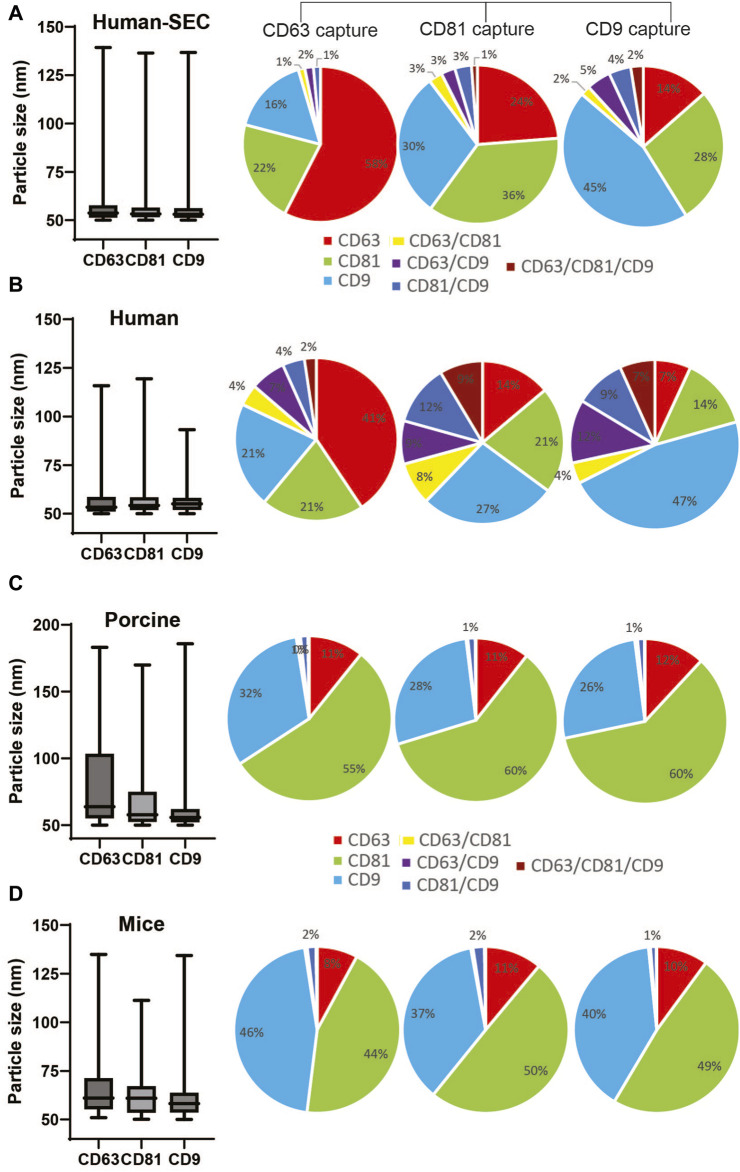
Comparison of particle size and marker composition of EVs isolated by SEC from human serum or by NTI-EXO from human, porcine, or mouse sera. The ExoView R200 platform was used for characterization. **(A)** Particle size and marker composition from SEC-isolated human EVs. NTI-EXO EV isolation results from **(B)** human serum, **(C)** porcine serum, and **(D)** mouse serum. The NTI- or SEC-isolated EVs were characterized for detection of CD81, CD63, and CD9 and the isotype control MIgG. Co-localization of serum-derived EVs was shown using 3 fluorescent channels and an overlay of fluorescent images. Data represents the CD81, CD63, and CD9 co-localization (%). Data are the mean ± SEM of 3 independent biological experiments with 3 technical replicates.

We routinely analyzed and monitored the particle size and concentration of isolated EVs using NTA. However, we noticed inconsistency in the results using this technology. Thus, to further validate the integrity and distribution of markers from NTI-EXO-isolated EVs, we used the ExoView R200 system. The ExoView R200 characterizes vesicle count, size, and surface markers to measure the sizes of EVs as small as 50 nm. NTI-EXO-isolated EVs from human, porcine, and mouse serum (Figure 1) were compared with human EVs purified using SEC (Supplementary Figure S1). For the assay, isolated EVs were immunologically immobilized on the pre-coated tetraspanin panel chip coated with anti-CD9, -CD63, and -CD81 antibodies. This enabled us to accurately determine the size of EVs (50–70 nm) as well as the distribution and co-localization of CD9, CD63, and CD81. As shown in Figure 5A, the mean size of SEC-isolated pure EVs captured by anti-CD63, -CD81, and -CD9 antibodies was 63 nm, 59 nm, and 57 nm, respectively. Interestingly, 58% of CD63-positive EVs were found on CD63 capture spots that were also CD81^−^and CD9-positive. The high percentage of CD63 on EVs was consistent with the observed immunoblotting results (Figure 1C). Between 1% and 3% of the SEC-isolated EV population was detected by 2 or 3 markers together. For the NTI-EXO method, the co-localization of 2 or 3 markers on single particle, CD63/CD81, CD63/CD9, CD81/CD9, and CD63/CD81/CD9, ranged from 4% to 10% (Figures 5A,B).

Isolated porcine EVs captured by anti-CD63, -CD81, and -CD9 antibodies had a mean particle size of 70 nm, 63 nm, and 63 nm, respectively. Of note, NTA data suggests a particle size range of 100–140 nm (Figures 2B,D). The overall distribution of CD81, CD9, and CD63 markers from captured EVs was 60%, 30%, and 11%, respectively (Figure 5C). The distribution of CD63^−^, CD81^−^, and CD9-positive mouse-derived EVs was more similar to that of porcine EVs (Figures 5C,D) when compared to human EVs. The mean size of mouse-derived EVs captured by anti-CD63, -CD81, and -CD9 antibodies was 62 nm, 65 nm and 61 nm respectively. The distribution of CD81 and CD9 was around 45% of the population, whereas that of CD63 was close to 10%. Of note, all 3 anti-tetraspanin antibodies worked successfully for human, porcine, and mouse samples, suggesting cross-reactivity. Overall, when compared to SEC, NTI-EXO resulted in better yields with consistent integrity and high quality. Based on all the above the characterization results, we did not attempt to pass isolated EVs through a 0.22 µm filter since the further clarification was not necessary.

### Investigating NTI-EXO applicability to monitor lung transplant patients with chronic rejection

**FIGURE 6:**
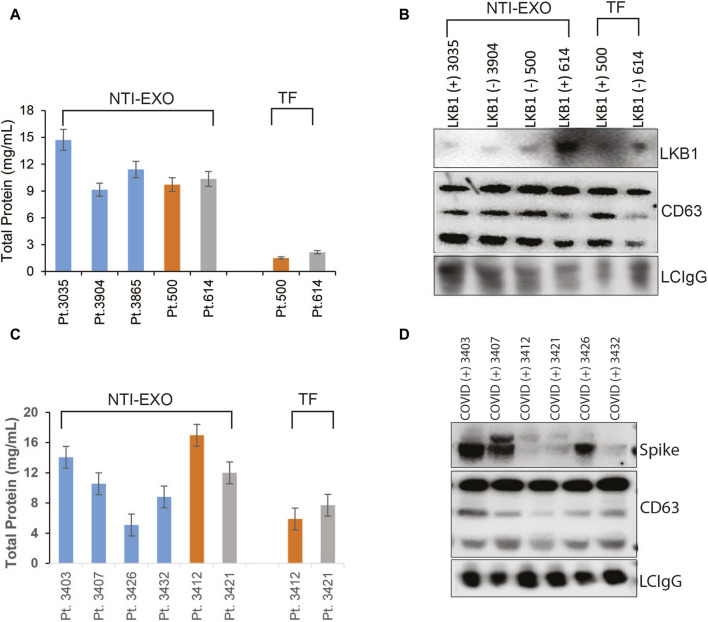
Validation of NTI-EXO by comparison with our previous disease marker results. Previous work with UC and commercial reagents was reproduced using the NTI-EXO EV isolation method. Frozen patient plasma samples were treated with NTI-EXO to isolate EVs. EVs isolated from a lung transplant patient sample: **(A)** total recovery, and **(B)** characterization using immunoblotting for LKB1 (Santa Cruz Biotechnology and Cell Signaling), CD9, and a loading control LC-IgG. **(C, D)** EVs isolated from plasma from a lung transplant patient with COVID-19 using NTI-EXO. Extracted EV samples were immunoblotted for SARS-CoV-2 spike protein, CD63, with loading control LCIgG. Disease markers as well as EV markers were detected in EV-enriched proteins isolated by TF or NTI-EXO.

After successful characterization of NTI-EXO-isolated EVs for quality and integrity, we investigated the applicability of the method to monitor chronic rejection markers in samples from lung transplant patients. Our group is actively studying and monitoring EV-based markers to predict failure or long-term survival of organ transplants (Bansal, Sharma et al., 2018; Ravichandran, Bansal et al., 2019; Bansal, Limaye et al., 2021; Bansal, Perincheri et al., 2021; Bansal, Tokman et al., 2021; Rahman, Ravichandran et al., 2023). For case study one, we used samples from a study that determined a loss of liver kinase B1 (LKB1) in circulating EVs isolated from lung recipients with chronic lung allograft dysfunction (Rahman, Ravichandran et al., 2022; Rahman, Ravichandran et al., 2023). Five plasma samples were processed for EV isolation by the NTI-EXO method. As shown in Figure 6A, from 200 µL of plasma, we recovered 9–15 mg/mL total protein using the NTI-EXO method, whereas the commercial TF kit yielded 2–3 mg/mL. Twenty micrograms of total protein was resolved for immunoblotting. As shown in Figure 6B, LKB1 along with CD63 and the loading control LC-IgG, was successfully detected from EVs isolated from a COVID-positive lung transplant patient. For case study two, we investigated the detection of the SARS-CoV-2 spike protein in EVs isolated by NTI-EXO from a lung recipient with COVID-19, which triggered chronic rejection (Gunasekaran, Bansal et al., 2020; Bansal, Perincheri et al., 2021). Of note, the SARS-CoV-2 spike protein is often detected in EVs isolated from infected patients. The commercial TF reagent yielded 4–6 mg/mL of total protein. For the same volume of plasma, NTI-EXO recovered 10–15 mg/mL of protein, a 2- to 3-fold improvement (Figure 6C). EV isolation followed by immunoblotting showed most of the samples were positive for the SARS-CoV-2 spike protein, as predicted earlier (Bansal, Tokman et al., 2021). These EVs also showed consistent detection of CD63 as well as the loading control LC-IgG (Figure 6D). We also observed that the EV pellet from samples from infected patients was visually larger and contained higher EV yields than the pellet from non-infected patients. Together, these two independent case studies verified the applicability of NTI-EXO for clinical samples, which benefited from enhanced recovery without compromising the exosomal integrity.

### A drop method to isolate EVs from small volumes of serum

EVs isolated from mice are providing meaningful insights for several disease models; however, the major limiting factor is the volume of serum available from mice. In our laboratory, 25 g mice which have 2.0 mL blood can provide 0.2 mL of blood, which yields 100–120 µL of serum, for survival studies. Of note, if an experiment involves collection of blood at different time points, then the approved method is to draw 2 drops of blood, which yields roughly 50 µL of serum. For our model of lung, kidney, and heart transplant, 1 mouse can provide 100 ± 30 µL of serum after sacrifice, which yields 60–100 µg of protein (using the commercial TF or UC method). In this scenario, serum collected from 1 mouse gives enough protein/EVs for only 4 to 5 western blots. For marker discoveries, we have often used 4 to 5 mice due to the limited yield of exosomal proteins per mouse. Encouraged by the high recovery yields and exosomal integrity achieved from the NTI-EXO method, we applied this method to the very small samples and yielded 3- to 5-fold more exosomal proteins.

**FIGURE 7:**
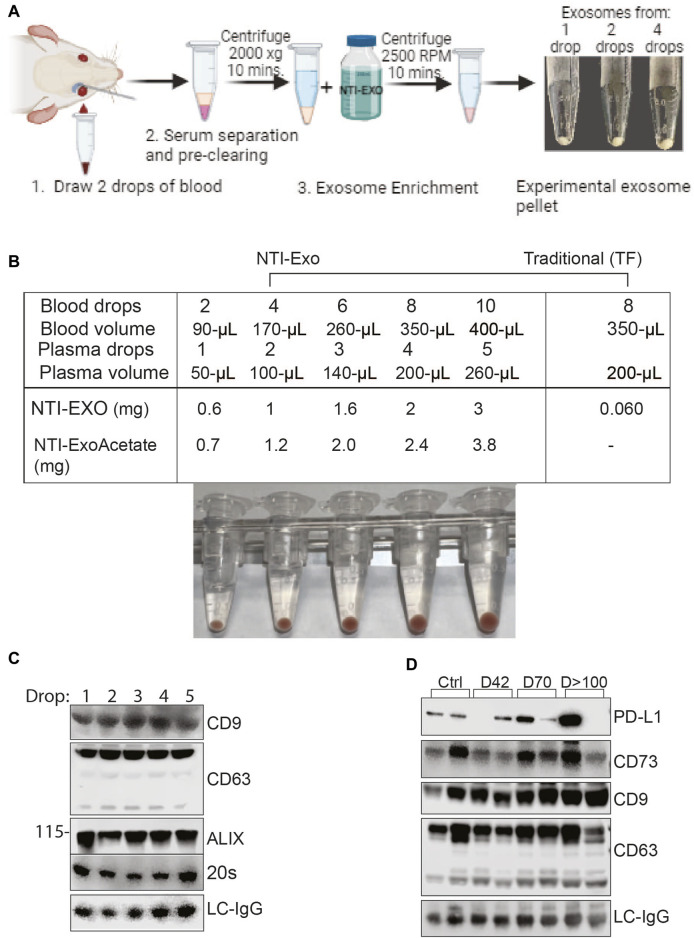
Breakthrough single drop-method to isolate EVs from mouse serum. **(A)** Depiction of method to isolate EVs from drop-sized samples to obtain a visible pellet of EVs. **(B)** Data from 2 independent experiments of porcine or mouse sera obtained from animals undergoing organ transplant studies. EVs were isolated from fresh serum or frozen plasma samples by NTI-EXO or TF. The NTI-EXO-Acetate method increased recovery. Visible pellets can be seen in the samples processed by the NTI-EXO method. **(C)** At 12 h of EVLP, porcine plasma was assayed for EV markers. From 1 to 5 drops of plasma were acquired and treated with NTI-EXO for EV isolation followed by protein measurement and immunoblotting for EV markers including CD9, CD63, ALIX, 20 s, and the loading control LC-IgG. Twenty micrograms of total protein were resolved for immunoblotting. **(D)** Two drops of plasma from kidney transplanted mice were used for EV isolation to monitor PD-L1, CD73, EV markers CD9 and CD63, and the loading control LC-IgG. Data suggest small-sized serum samples can be used successfully to characterize disease-related markers.

To obtain the miniature sized samples, retro-orbital bleeding of anesthetized mice was carried out by puncturing the sinus to collect one to three drops of blood per mouse (Figure 7A). Samples were quickly processed to recover serum followed by storage or EV extraction. In our practice, 2 drops of blood (roughly 100-µL) recovered 50–70 µL of serum, and NTI-EXO extraction recovered about 0.6 mg of EV-enriched proteins. As shown in Figure 7A, isolated EV pellets are visible from 1-, 2-, and 4-drop samples. Having confirmed consistency, we explored the NTI-EXO drop method using samples from our ongoing porcine model of lung transplant studies. Blood was collected from *ex-vivo* lung perfusion experiments, and 1 to 5 drops of serum was subjected to 3 independent assays: commercial TF, NTI-EXO, and NTI-EXO-Acetate (see Figure 2). The traditional precipitation method recovered 60 µg of total protein from 200 µL of serum, whereas NTI-EXO extracted 2 mg from the same volume (Figure 7B). More strikingly, this recovery was further improved by 20%–25% using the NTI-EXO-Acetate method. When 2 drops of porcine plasma were processed for EV isolation, the method yielded 0.6 and 1 mg of total protein. From this sample, 20 µg of protein were immunoblotted and EV markers ALIX, CD9, 20 s, and CD63 were detected (Figure 7C). In consistency checks, the same batch or source of serum showed a 4%–5% standard error of the final yields.

After developing consistency in the EV isolation process, we used the NTI-EXO-drop method to monitor kidney transplant-related chronic immune injury. We have found in a mouse model (BALB/c; H2d to C57BL/6; H2b) that allogenic transplant-related inflammatory changes develop on day 7 and then decrease by post-transplant day 42, and moderate interstitial fibrosis and tubular atrophy occurred during chronic immune injury on day >100. Circulating EVs play an important role in the activation of immune responses to kidney self-antigens leading to the immune pathogenesis (Itabashi, Ravichandran et al., 2022). Exosomal PD-L1 suppresses T cell function in cancer models (Poggio, Hu et al., 2019) and also induces T cell injury (Bracamonte-Baran, Florentin et al., 2017). Extracellular vesicles containing CD73 have the ability to suppress Th1 CD4^+^ T cell responses (Smyth, Ratnasothy et al., 2013; Agarwal, Fanelli et al., 2014). As shown in Figure 7D, along with EV markers CD9 and CD63, we detected CD73 and PD-L1 in stages of transplant-related chronic immune injury. This data is in good agreement with a previous report (Ravichandran, Itabashi et al., 2022). In the above case study experiments, the recovery of EVs was consistent and 5-fold higher than commercial methods. Altogether, our NTI-EXO, NTI-EXO-Acetate, and NTI-EXO-drop methods delivered consistent recovery yields with high integrity (i.e., marker protection). The single drop method will be especially useful for small animal (e.g., mouse) models.

## Discussion

In this study, three modified gentle precipitation-based methods were used to enrich EVs from human, porcine and mice serum samples with consistency, integrity, and high recovery: NTI-EXO, NTI-EXO-Acetate, and NTI-EXO-drop. Notably, EV isolation methods using commercial kits, precipitation reagents, SEC, and UC still face many challenges, including recovery, consistency, structural integrity, size, and cost. In a series of experiments, we evaluated the NTI-EXO method on different plasma and serum samples and compared the results to those of traditional UC and SEC methods. First, we optimized several conditions depicted in Figure 1 to enrich for high yields and purity of EVs. We validated quality and integrity using NTA, ExoView, SDS-PAGE, TEM and immunoblotting (Figures 2–5). Based on quality control criteria, we intend to utilize this method for our ongoing research projects which include human, porcine, and mouse blood samples. Ultimately, we refined a technique for extracting EVs from the equivalent of 2 drops of blood, a crucial development particularly applicable to small animal studies. In a routine 3-step, 1-h protocol, we recovered 1 mg of EV-enriched protein from 2 drops of blood (Figure 7). To add, plasma and serum samples were processed for EV isolation in an hour without the need for subsequent purification. The reagent can be lab prepared and has a long shelf-life, good stability, acceptable levels of contamination; it is also cost effective. Since we have proven that the NTI-EXO method is reliable, quick, and consistent, we plan to repack animal-derived exosomes to use as a drug delivery vehicle in the near future. One of the limitations of our current method is that we did not characterize other potential markers associated with other exosome contents.

Moreover, characterization of isolated exosomes still face several criticisms and often requires further validation using orthogonal techniques (Gandham, Su et al., 2020) i.e., NTA, flow cytometry, dynamic light scattering, and electron microscopy (Varga, Yuana et al., 2014; Szatanek, Baj-Krzyworzeka et al., 2017). However, in addition to NTA technology (Figure 2), we used ExoView R200, a new generation EV characterization technique, to validate the size and the integrity of the NTI-EXO-isolated EVs (Figure 5). For the ExoView assay, samples were placed onto a chip coated with anti-tetraspanin antibodies to capture EVs followed by detection with fluorescent antibodies to measure surface protein expression levels. ExoView R200 offers a more convenient and streamlined solution for EV characterization than most other methods (Lai, Chau et al., 2022). ExoView data provided a more accurate size measurement of EVs 55 nm–80 nm than NTA which was 100 nm–250 nm. Although NTA is more convenient, user friendly and cost effective for daily use which serves the purpose, however its overestimation of particle diameter to 2-fold is known (Bachurski, Schuldner et al., 2019). In this regard, we used ExoView to validate our data. For comparison, our data suggested that EVs isolated by NTI-EXO or SEC from human, porcine, and mouse serum samples were very similar in particle size (Figure 5). Of note, the EVs isolated from human, porcine, and mouse sera were consistent for particle size (55–65 nm) and positivity for CD9, CD81, or CD63 markers.

NTI-EXO presents advantages over conventional methods currently in use for isolating EVs. Among the prevalent EV isolation techniques—such as SEC, salt precipitation, microfiltration, and UC—UC is widely embraced in research due to its reproducibility, despite its limitations in handling high sample volumes and requiring specific expertise (Patel, Khan et al., 2019; Sidhom, Obi et al., 2020). Furthermore, UC and pressure-based steps can potentially damage and negatively impact protein and RNA contents (Lamparski, Metha-Damani et al., 2002). PEG-based methods have long been used for EV isolation with other techniques (Rider, Hurwitz et al., 2016; Kalarikkal, Prasad et al., 2020; Zhang, Borg et al., 2020; Yakubovich, Polischouk et al., 2022). Our current precipitation method, compared to prior findings, proves more efficient when working with smaller sample sizes. Dealing with limited sample sizes is a significant challenge across almost all isolation methods. Addressing this constraint, the newly developed NTI-EXO reagent can extract EVs from just a drop of blood. This advancement is particularly advantageous for studies involving small animals and infants, also reducing the necessity for arterial blood draws from enrolled patients in studies. While our study primarily focused on isolating EVs from plasma and serum samples, this methodology can be extended to various other biological fluids.

## Data Availability

The original contributions presented in the study are included in the article/Supplementary Material, further inquiries can be directed to the corresponding author.

## Abbreviations

BCA, Bicinchoninic acid; BCG Bromocresol Green; DLS Dynamic Light Scattering; EDTA Ethylenediaminetetraacetic acid; EV Extracellular vesicle; MHC Major Histocompatibility molecules; NTI Norton Thoracic Institute; PBS Phosphate-buffered saline; PEG polyethylene-glycol; sEV Small EV; SEM Standard error of the mean; SEC Size exclusion chromatography; RIPA Radio-immunoprecipitation assay; LTX Lung transplant; TBS-T Tris-buffered saline with 0.1% Tween 20; TF ThermoFisher kit; UC Ultracentrifugation.
