# Supplementary material for: Enhanced enrichment of extracellular vesicles for laboratory and clinical research from drop-sized blood samples
Source: Front Mol Biosci. 2024 Aug 15;11:1365783. doi: 10.3389/fmolb.2024.1365783 (PMC11358096; doi:10.3389/fmolb.2024.1365783)

Figure supplementary 3: Full western blots

Figure 1D.

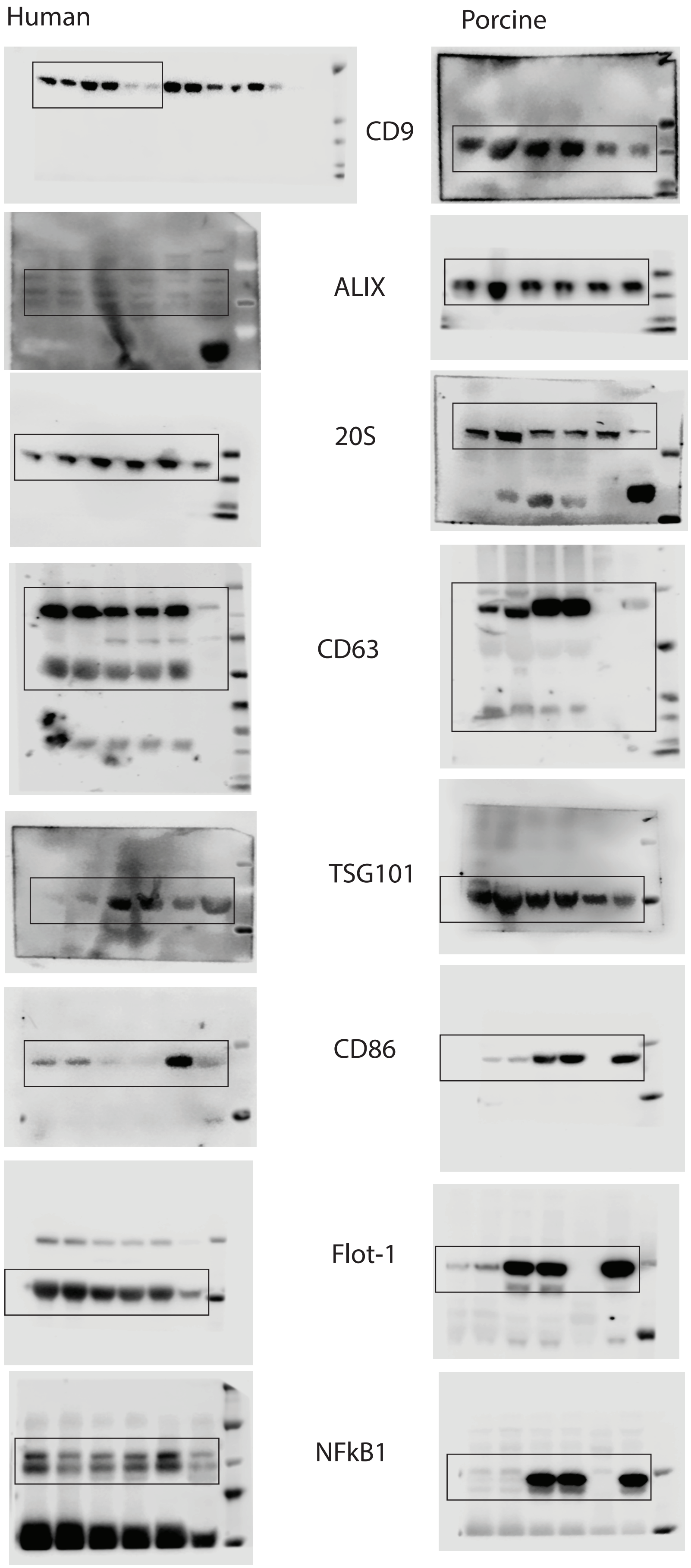

Figure 4D.

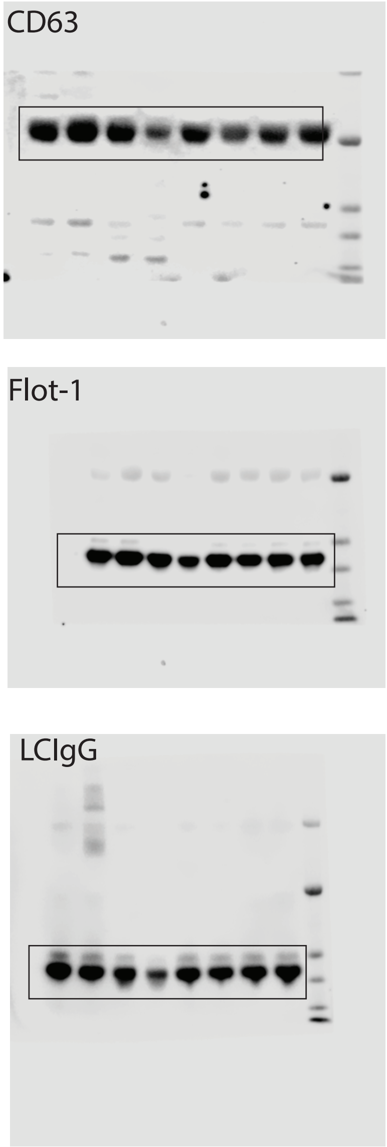

Figure 6B.

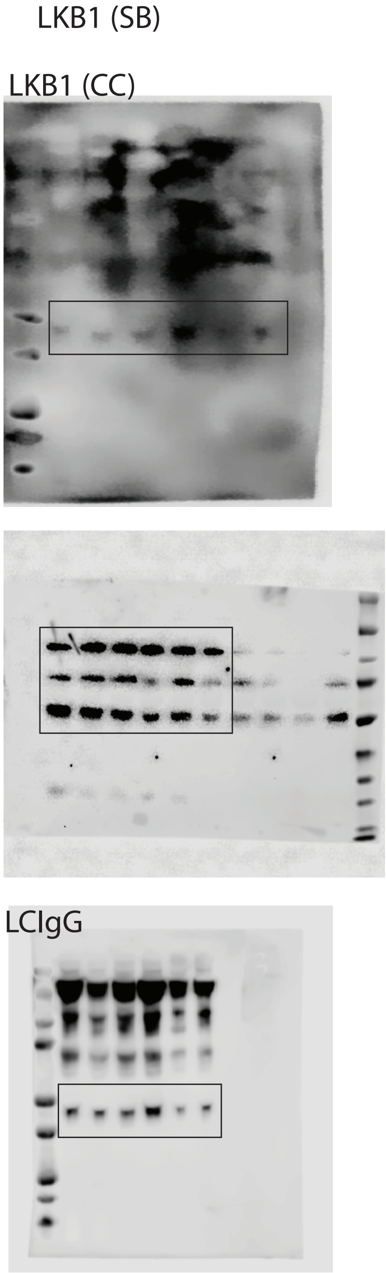

Figure 6D.

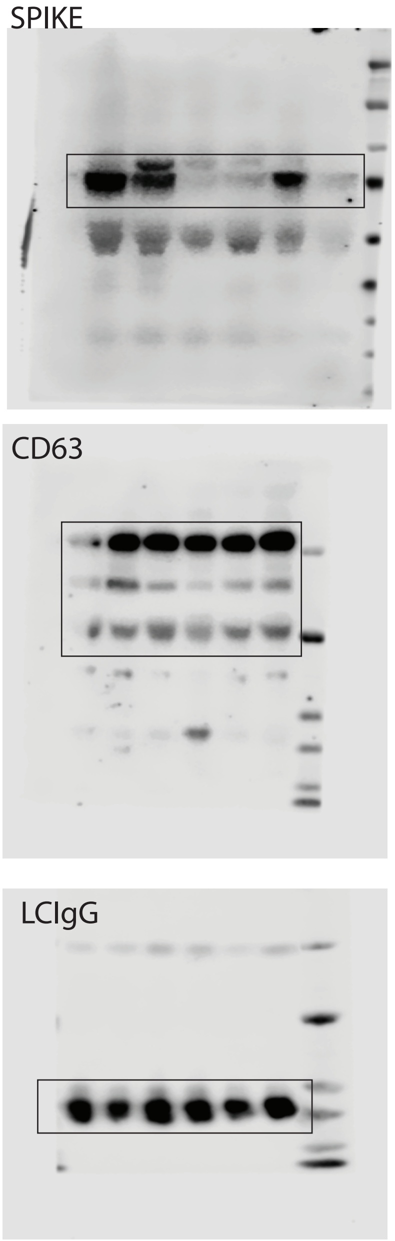

Figure 7. C

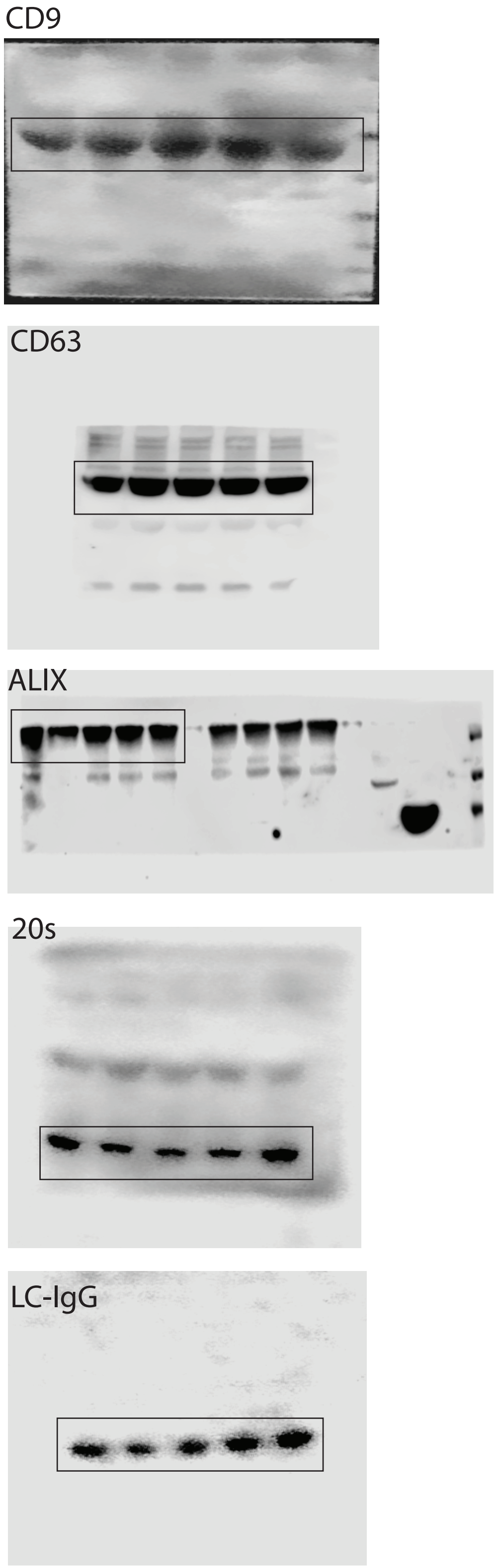

Figure 7. D

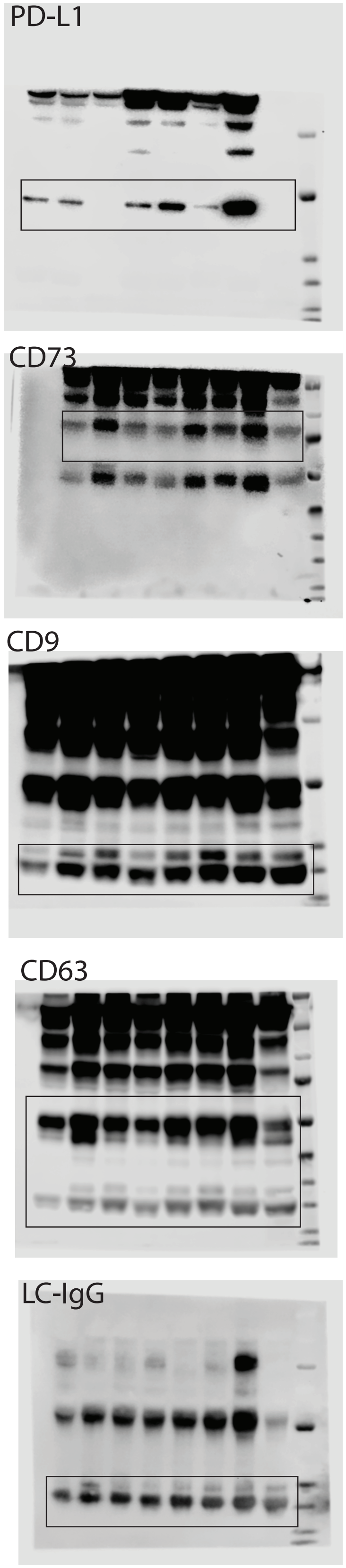

Figure 3H

20s

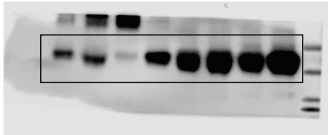

TSG101

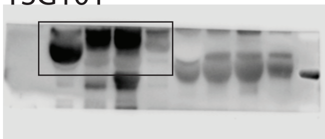

TSG101

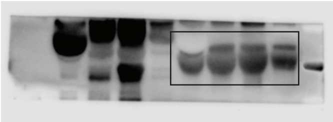

ApoA1

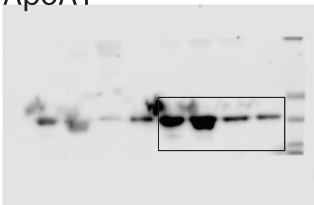

ApoA1

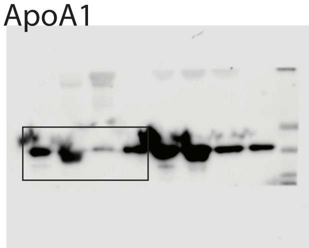

Albumin

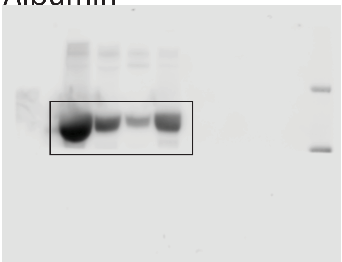

Albumin

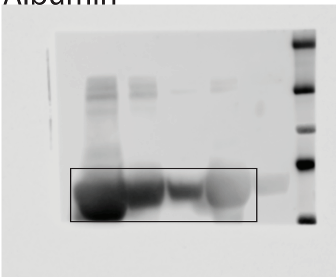

IgG-LC

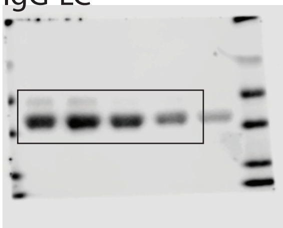

IgG-LC

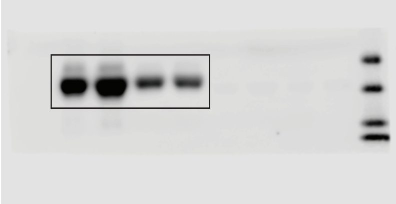

Figure 3B SDS-PAGE

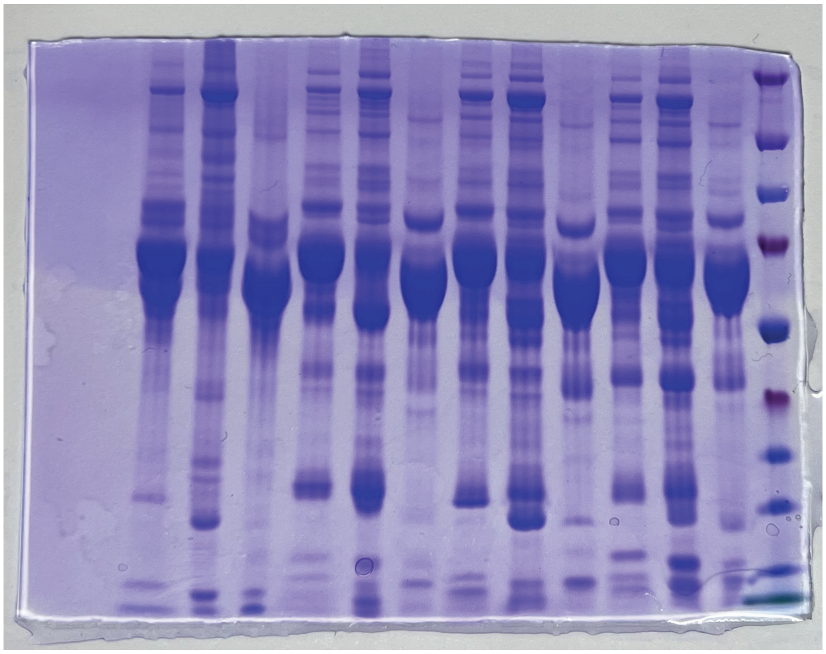

Supplement: Supplementary file 1 [file DataSheet2.PDF]
