# Supplementary material for: Enhanced enrichment of extracellular vesicles for laboratory and clinical research from drop-sized blood samples
Source: Front Mol Biosci. 2024 Aug 15;11:1365783. doi: 10.3389/fmolb.2024.1365783 (PMC11358096; doi:10.3389/fmolb.2024.1365783)
Supplement: Supplementary file 2 [file DataSheet1.PDF]

Enhanced enrichment of extracellular vesicles for laboratory and clinical research from drop-sized blood samples

Alexa Guerrero-Alba, Sandhya Bansal, Aryan N Sankpal, Geetanjali Mitra, Mohammad Rahman, Ranjithkumar Ravichandran, Christin Poulson, Timothy P. Fleming, Michael A. Smith, Ross M. Bremner, T. Mohanakumar and Narendra V. Sankpal*

Figure S1.

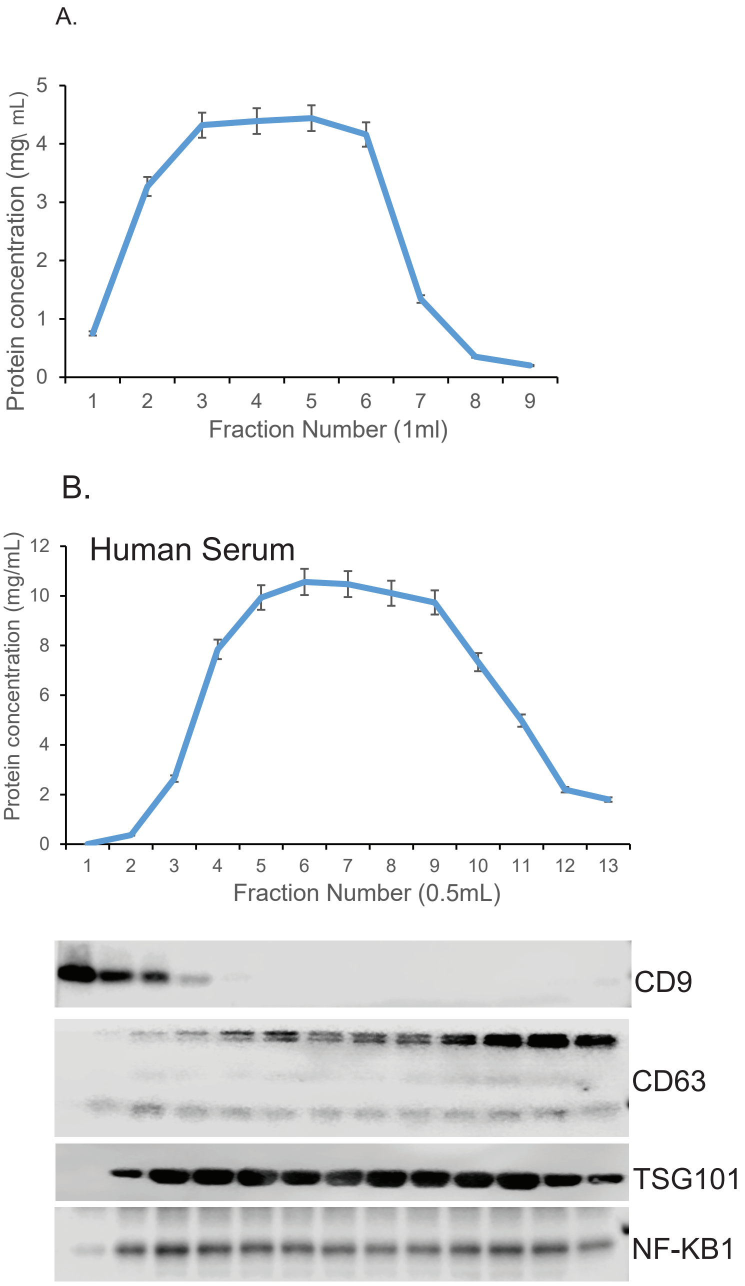

Figure supplementary 1. Purification and characterization of exosomes isolated from human serum using size exclusion chromatography (SEC).
Two independent experiments were performed with 1.0 mL of serum. Fractions, (A) 1.0 mL and (B) 0.5 mL, were collected and analyzed for total protein. Collected fractions were immunoblotted to detect CD9, CD63, TSG101, and NK-kB.

Figure S2.

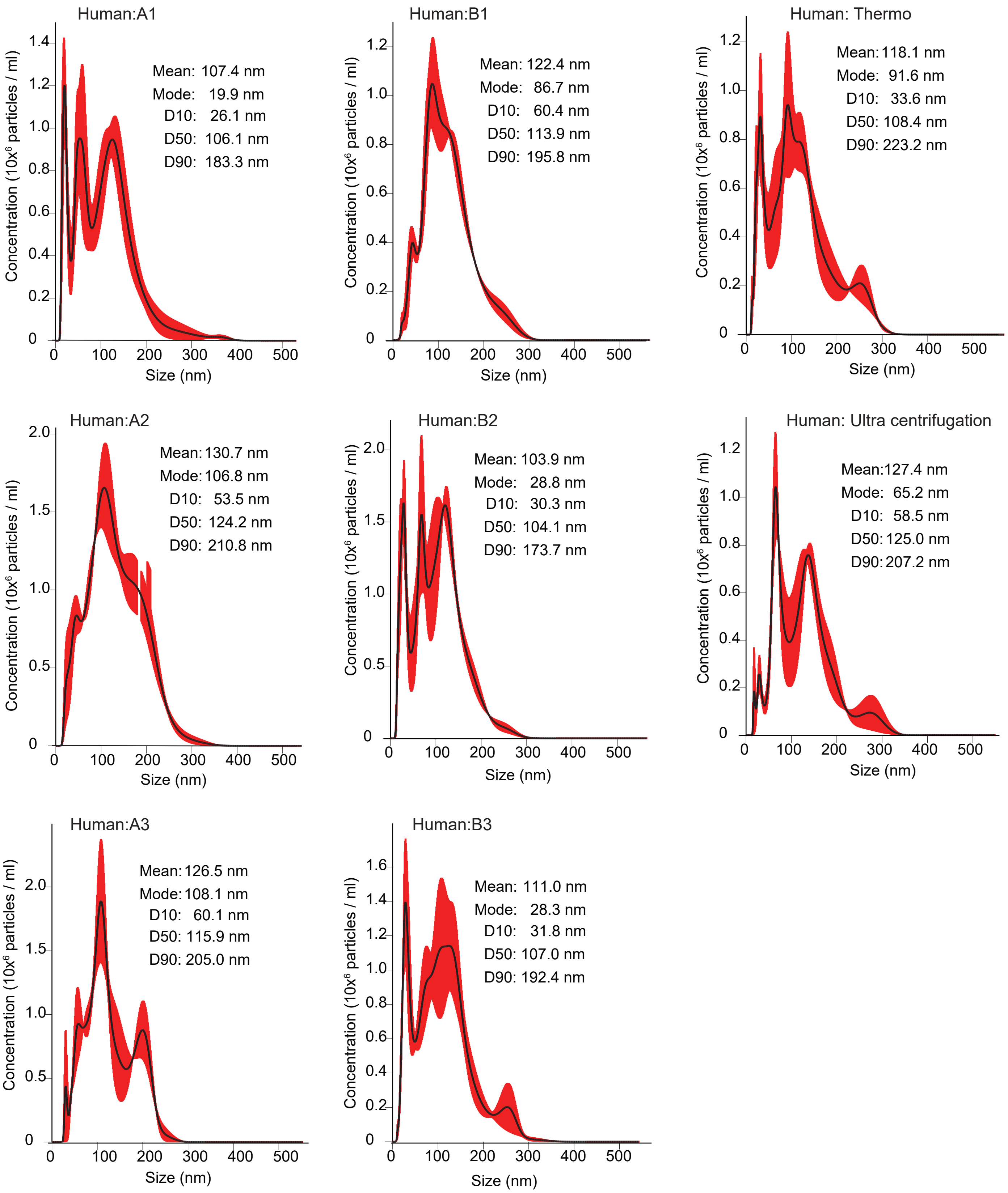
Human:A1
Mean: 107.4 nm
Mode: 19.9 nm
D10: 26.1 nm
D50: 106.1 nm
D90: 183.3 nm
Human:B1
Mean: 122.4 nm
Mode: 86.7 nm
D10: 60.4 nm
D50: 113.9 nm
D90: 195.8 nm
Human: Thermo
Mean: 118.1 nm
Mode: 91.6 nm
D10: 33.6 nm
D50: 108.4 nm
D90: 223.2 nm
Human:A2
Mean: 130.7 nm
Mode: 106.8 nm
D10: 53.5 nm
D50: 124.2 nm
D90: 210.8 nm
Human:B2
Mean: 103.9 nm
Mode: 28.8 nm
D10: 30.3 nm
D50: 104.1 nm
D90: 173.7 nm
Human: Ultra centrifugation
Mean: 127.4 nm
Mode: 65.2 nm
D10: 58.5 nm
D50: 125.0 nm
D90: 207.2 nm
Human:A3
Mean: 126.5 nm
Mode: 108.1 nm
D10: 60.1 nm
D50: 115.9 nm
D90: 205.0 nm
Human:B3
Mean: 111.0 nm
Mode: 28.3 nm
D10: 31.8 nm
D50: 107.0 nm
D90: 192.4 nm
Concentration (10x6 particles / ml)
Size (nm)

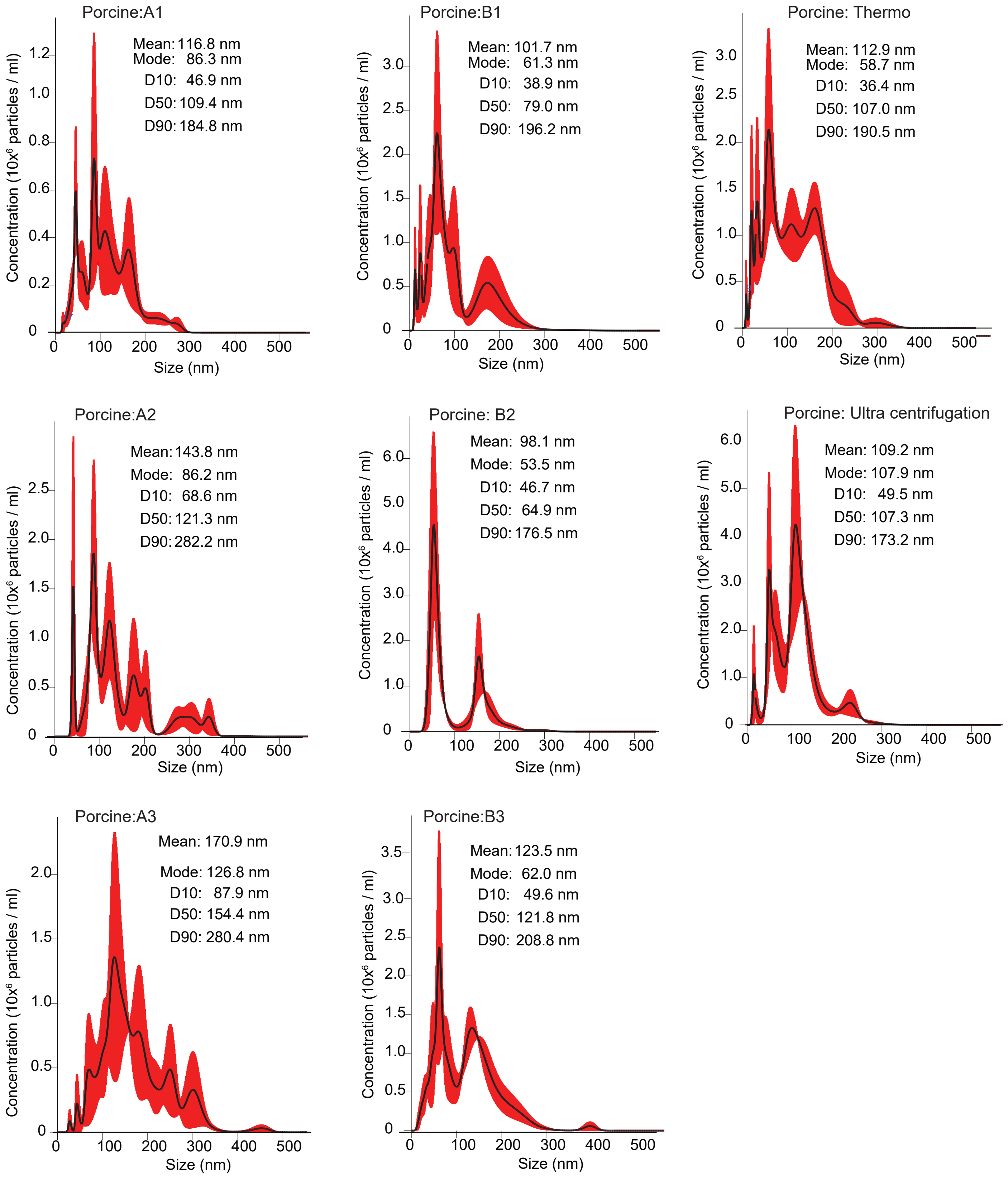

Figure supplementary 2: NanoSight data representing particle size of NTI-EXO-isolated EVs.
